# COVID-19 is Associated with a Lipid Storm that Worsens in Cases of Severe Pneumonia

**DOI:** 10.3390/microorganisms13112622

**Published:** 2025-11-19

**Authors:** Amani Bouabdallah, Mohamed Kacem Ben-Fradj, Mohamed Bessem Hammami, Rim Abdelmalek, Haifa Sanhaji, Timothée Klopfenstein, Moncef Feki

**Affiliations:** 1 Faculty of Medicine of Tunis, University of Tunis El Manar, Jebbari, Tunis 1007, Tunisia; kacimbf@gmail.com (M.K.B.-F.); monssef.feki@gmail.com (M.F.); 2 Laboratory of Biochemistry, Rabta Hospital, LR99ES11, Jebbari, Tunis 1007, Tunisia; 3 Service of Infectious Diseases, Rabta Hospital, Jebbari, Tunis 1007, Tunisia; 4 Department of Infectious Diseases and Tropical Medicine, Hôpital Nord-Franche-Comté, 90400 Belfort-Montbéliard, France; 5 Clinical Research Unit, Hôpital Nord-Franche-Comté, 90400 Belfort-Montbéliard, France

**Keywords:** Coronavirus, COVID-19, endocannabinoids, fatty acids, lipid mediators, lipid storm, oxylipins, SARS-CoV-2

## Abstract

Severe Coronavirus disease 2019 (COVID-19) is associated with abnormal innate and adaptive immune responses, as well as systemic alterations, including a shift in lipid network. A case–control study was conducted to describe the systemic lipidomic profile in COVID-19 according to disease severity. Selected polyunsaturated fatty acids (PUFAs), oxylipins, and endocannabinoids were analysed using a targeted liquid chromatography coupled to mass spectrometry in tandem method. Multivariate receiver operating characteristic curve-based model evaluation was performed to define a lipidomic signature for the disease. A total of 135 hospitalized COVID-19 patients, of whom 85 had severe form, and 134 healthy individuals were included. Patients exhibited increased levels of free PUFAs, proinflammatory and pro-resolving oxylipins, and endocannabinoids compared to controls. A combination of five lipid mediators, i.e., prostaglandin D2, prostaglandin E2, thromboxane B2, lipoxin B4, and 2-archidonylglycerol, discriminates patients from control individuals with excellent accuracy [AUC, 0.977 (0.950–0.995)]. The severe form is characterized by an imbalance between proinflammatory and pro-resolving oxylipins and increased endocannabinoids. COVID-19 is associated with a lipid storm that conditions disease severity. Targeting lipid mediators-related metabolic and signalling pathways could be an interesting therapeutic option in severe forms.

## 1. Introduction

Coronavirus disease 2019 (COVID-19) is a viral infection caused by the single-stranded RNA virus, severe acute respiratory syndrome coronavirus 2 (SARS-CoV-2), belonging to the family *Betacoronavirusidae*. The disease has been the deadliest pandemic of the 21st century so far, causing more than 7.1 million deaths worldwide [1]. Severe forms of the infection manifest with a life-threatening systemic and uncontrolled inflammation, causing acute respiratory distress and multiorgan failure [2], needing hospitalization and specific care. Patients with the severe form present with an abnormal immune landscape, characterized by an overactivated innate immune response and an impaired adaptive immune response [3]. It involves overproduction of various potent inflammatory mediators, including cytokines, chemokines, complement factors, and lipid mediators (LMs) [3]. Therefore, therapeutic strategies that target the immune system and inflammatory response are likely to be a suitable approach for recovery from COVID-19.

Polyunsaturated fatty acids (PUFAs) are key dietary factors and precursors of a variety of LMs, including oxylipins and endocannabinoids (eCBs) [4]. These bioactive compounds exert a large array of physiological functions, including inflammation, immunity, and haemostasis [4]. Oxylipins, generated by the enzymatic oxidation of PUFAs by cyclooxygenases (COX), lipoxygenases (LOX), and cytochrome P450 (CYP) enzymes, intervene in the initiation, triggering, and resolution of inflammation [5]. The n6 arachidonic acid (AA) and deriving prostaglandins (PGs) and leukotrienes (LTs) are generally pro-inflammatory/prothrombotic mediators [4], whereas AA-derived lipoxins (LXs) are pro-resolving mediators. The n3 PUFAs eicosapentaenoic (EPA), docosapentaenoic (DPA), and docosahexaenoic (DHA) acids and n3-derived oxylipins (i.e., resolvins (RVs), protectins (PDs), and maresins) are anti-inflammatory/pro-resolving mediators [4,6]. LXs, RVs, PDs, and maresins are grouped under the term specialized pro-resolving mediators (SPMs) [7]. Another group of signalling lipids, produced by the enzymatic hydrolysis of cell membrane phospholipids, eCBs are the endogenous ligands of endocannabinoid receptors. They are part of the endocannabinoid system (ECS), which also involves enzymes that synthesize and degrade eCBs, transporters for cellular uptake, and receptors. The system plays key roles in neurotransmission, immune modulation, and regulation of the inflammatory response, among other functions [8].

Extensive research has highlighted hyperactivity of the immune system and enhanced inflammatory response in COVID-19. Most studies have focused on cytokines, chemokines, soluble proteins, and immune cells [9]. Studies examining LMs in this infection revealed an altered lipidomic profile in patients [10,11,12,13,14,15,16,17,18,19,20,21,22,23,24]. The findings are inconsistent across studies, particularly regarding the association with disease severity. Most studies have focused on eicosanoids, or the canonical 2-arachidonoylglycerol (2-AG) and anandamide (AEA), involved small sample sizes, lacked control for potential confounding factors, such as body composition and medication, and have been mainly conducted in European and Asian populations. They could be considered pilot studies requiring further validation. Moreover, the protocols used for LMs analysis, including mass spectrometry-based methods, are varied [25], making comparisons between studies quite challenging.

In Tunisia, a North African country of nearly twelve million inhabitants, more than one million people have been infected with SARS-CoV-2, and around 30,000 died from COVID-19 during the pandemic [26]. Abundant research has described the epidemiological features of COVID-19 in Tunisia [27,28], but no lipidomic data have been reported for Tunisian patients. The current study aimed to (1) determine the circulating profiles of PUFAs, oxylipins, and eCBs in hospitalized COVID-19 patients, (2) identify biomarkers and lipidomic signatures for the disease, and (3) examine the association of these metabolites with disease severity and specific conditions.

## 2. Materials and Methods

### 2.1. Study Design

A case–control study was conducted at Rabta Hospital (Tunis, Tunisia) between October 2020 and June 2021, involving hospitalized COVID-19 patients and healthy controls (HC). All patients admitted to the infectious diseases department during the study period were included. Patients under eighteen, having received treatment for COVID-19 (corticosteroids, antibiotics), having renal or liver failure, and pregnant and breastfeeding women were not included. Patients discharged, transferred, or deceased within 24 h of admission were excluded. Patients were diagnosed with COVID-19 based on a positive quantitative polymerase chain reaction using pharyngeal swabs. Disease severity at admission was assessed according to the World Health Organization definition, i.e., oxygen saturation in room air < 90% or signs of severe respiratory distress (use of accessory muscles, inability to complete full sentences, respiratory rate > 30 breaths per minute) [29]. When the room air saturation value at admission was unavailable, the disease was considered severe for PaO_2_/FiO_2_ < 300 mm Hg. Controls were relatives of hospital employees with no known medical conditions, no symptoms, and showing a negative SARS-CoV-2 polymerase chain reaction test within the last 7 days. They were selected by matching patients by gender, five-year age class, and neighbourhood income. The ethics committee of Rabta Hospital approved the study protocol (protocol code, CEBM.EPS.HR/12 TM/2022). Written informed consent was obtained from all subjects involved in this study. This study was carried out under the ethics code of the world medical association (Declaration of Helsinki) for experiments involving humans.

### 2.2. Sample Collection and Lipid Mediators Profiling

Peripheral venous blood samples were collected in EDTA tubes after a 6 h fast, during the first 24 h of admission, before administering COVID-19 treatment. The specimens were immediately brought in ice boxes, and plasma was extracted after centrifugation. The samples were coded, de-identified, and stored at −80 °C until analysis. A targeted liquid chromatography coupled to mass spectrometry in tandem (LC-MS/MS)-based lipidomic was performed on the Nexera X2 system coupled to LCMS-8050 mass spectrometer (Shimadzu, Kyoto, Japan) using the Method Package for Lipid Mediators software (Version 2, Shimadzu). The details of the equipment and analytical procedures are described in a previous report [30]. The method allowed for assessing selected five PUFAs [i.e., α-linolenic acid (ALA), AA, EPA, DPA, DHA)], eighteen oxylipins [i.e., PGD2, PGE2, 6-keto-PGF1α, PGF2α, thromboxane (TX) B2 (TXB2), LTB4, LTD4, LTE4, LTF4, LXA4, 6-epi-LXA4, 15-epi-LXA4, LXB4, RVD1, RVD5, PDX, 7,17-di-OH-DPA, 18-hydroxyeicosapentaenoic acid (18-HEPE)], and seven eCBs [i.e., 2-AG, AEA, palmitoylethanolamide (PEA), oleoylethanolamide (OEA), α-linolenoylethanolamide (ALEA), eicosapentaenoylethanolamide (EPEA), and docosahexaenoylethanolamide (DHEA)].

### 2.3. Statistical Analysis

We utilized the online software package MetaboAnalyst 6.0 (https://www.metaboanalyst.ca (accessed on 28 September 2025) SPSS software version 22.0 (SPSS Inc., Chicago, IL, USA), and GraphPad Prism v9.0 software (GraphPad Software, San Diego, CA, USA) for statistical analyses and figures plotting. The data set was pre-processed; missing values for lipid mediators (<3%) were handled through the linear interpolation method using the SPSS software. The Shapiro–Wilk test was used to test the normality of continuous variables. Comparisons between groups were performed using the Chi-squared test, Fisher test, Student’s test, or Mann–Whitney test, as appropriate. Before the analysis with MetaboAnalyst, the dataset was log-transformed and auto-scaled. A volcano plot analysis was applied to test the differences in LMs between patients and controls. Receiver operating characteristic (ROC) curve analyses were performed to assess the effectiveness of biomarkers in distinguishing COVID-19 patients from controls. Partial least-squares discriminant analyses (PLS-DA) were performed to select and rank the metabolites based on their importance in differentiating patients and controls. Typically, the variable in projection (VIP) threshold used to identify significant variables is equal to or greater than one; the higher the threshold, the more significant the variable. We therefore chose a threshold of 1.5, which allowed for the selection of the most discriminant variables. Metabolites with a VIP value above 1.5 were introduced into a multivariate ROC curve-based model evaluation to identify a lipidomic signature for COVID-19. The performance of the model is evaluated using the area under the curve (AUC) with its 95% confidence intervals and its accuracy. According to Jones and Athanasiou’s criteria [31], an AUC of 0.75 to 0.92 indicates a “good accuracy”, 0.93 to 0.96 a “very-good” accuracy, and >0.97 an “excellent accuracy”.

## 3. Results

### 3.1. Participants and Disease Characteristics

One hundred thirty-four hospitalized COVID-19 patients and 135 healthy controls were included. Patients and controls did not differ according to age, gender, body composition, or tobacco smoking. In patients, disease symptoms dated back 10 days on average. At admission, asthenia, fever, dyspnoea, and myalgia were the most frequent symptoms. Of the 134 patients, 85 had a severe form (63.4%). Hypertension and diabetes were the most common comorbidities, affecting more than half of the patients (60% and 52%, respectively). There were no significant differences in age, gender, body composition, tobacco smoking, comorbidities, and drug use between non-severe and severe COVID-19 forms. The symptoms of fatigue and diarrhoea were more frequent in severe forms. Baseline respiratory rate and CRP, and D-dimer concentrations were higher, whereas SpO2 and the PaO_2_/FiO_2_ ratio were lower in patients with the severe form (Table 1).

### 3.2. Lipidomic Profile in COVID-19 Patients

**Figure 1:**
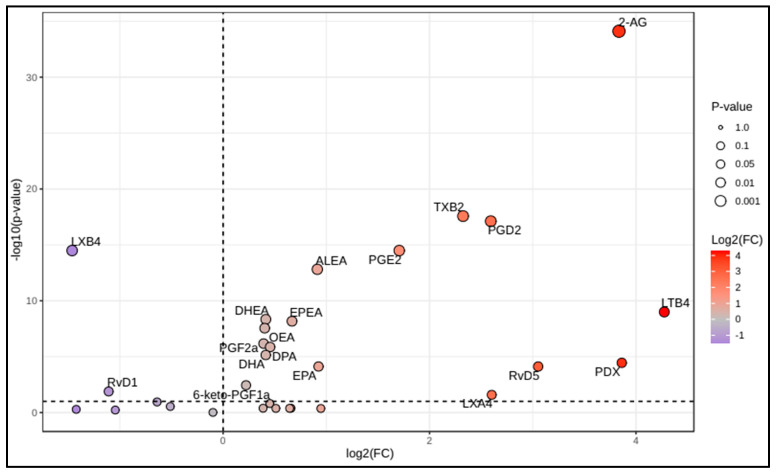
Volcano plot of selected polyunsaturated fatty acids, oxylipins, and endocannabinoids in Coronavirus disease 2019 (COVID-19) patients compared to controls.

**Figure 2:**
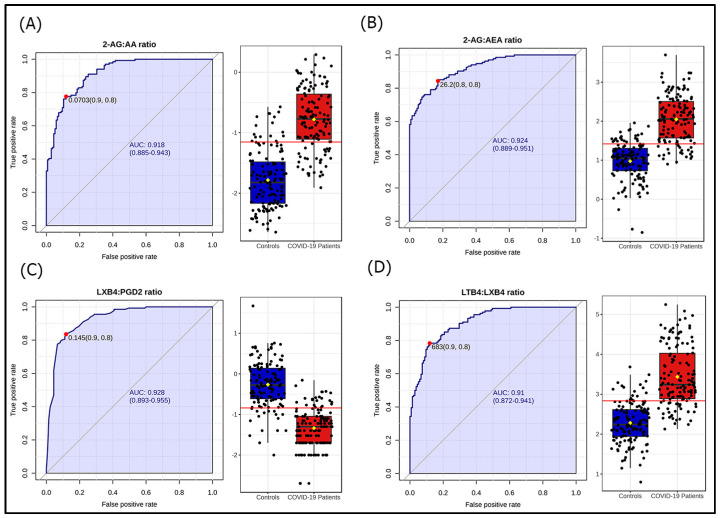
Operating receiver characteristic (ROC) curve and boxplot depiction of logarithmically transformed values of the ratios (**A**) 2-arachidonoylglycerol:arachidonic acid ratio (2-AG:AA ratio), (**B**) 2-arachidonoylglycerol:anandamide ratio (2-AG:AEA ratio), (**C**) lipoxin B4:prostaglandin D2 ratio (LXB4:PGD2 ratio), and (**D**) leukotriene B4:lipoxin B4 ratio (LTB4:LXB4 ratio) in Coronavirus disease 2019 (COVID-19) patients compared to controls. The red line in the box-plot representation corresponds to the logarithmically transformed cut-off value of the ROC curve (represented by a red point in the ROC curve).

Comparison between patients and controls revealed differences for 19 of the 30 LMs. COVID-19 patients had higher levels of n3 PUFAs (EPA, DPA, and DHA), proinflammatory (PGD2, PGE2, TXB4, LTB4, PGF2α, and 6-keto-PGF1α) and pro-resolving (PDX, RVD5, LXA4) oxylipins, as well as eCBs (2-AG, OEA, EPEA, and DHEA) (Table 2). Volcano plot analysis showed dissimilar LM distributions between patients and HC; the most significant differences were found for 2-AG and LTB4 (Figure 1). Univariate ROC analyses showed that PGD2:LXB4 ratio, LTB4:LXB4 ratio, 2-AG:AA ratio, and 2-AG:AEA ratio, differentiated COVID-19 patients with a “very good” accuracy (AUC > 0.90) (Figure 2).

The volcano plot of lipid mediators in COVID-19 patients compared to controls, with a fold change (FC) = 1, and a q-value < 0.1. The metabolites in the upper right quadrant are upregulated, those in the upper left quadrant are downregulated, while the metabolites below the horizontal dashed line are unchanged in patients compared to controls. The node size means the total number of metabolites in each cluster. The X-axis corresponds to log2(FC), and the Y-axis corresponds to −log10(*p*-value). Fold change (FC) is greatest for LTB4, PDX, and 2-AG (FC ≈ 15 to 20), and the difference between patients and controls is most significant for 2-AG (q-value < 10^−10^). 2-AG: 2-arachidonoylglycerol; 6-ketoPGF1a: 6-keto-prostaglandin F1α; ALEA: α-linolenoylethanolamide; DHA: docosahexaenoic acid; DHEA: docosahexaenoylethanolamide; DPA: docosapentaenoic acid; EPA: eicosapentaenoic acid; EPEA: eicosapentaenoylethanolamide; FC: fold change; LTB4: leukotriene B4; LXA4: lipoxin A4; LXB4: Lipoxin B4; OEA: oleoylethanolamide; (p): adjusted *p*-value; PDX: protectin DX; PGD2: prostaglandin D2; PGE2: prostaglandin E2; PGF2a: prostaglandin F2α; RVD1: resolvin D1; RVD5: resolvin D5; TXB2: thromboxane B2.

**Figure 3:**
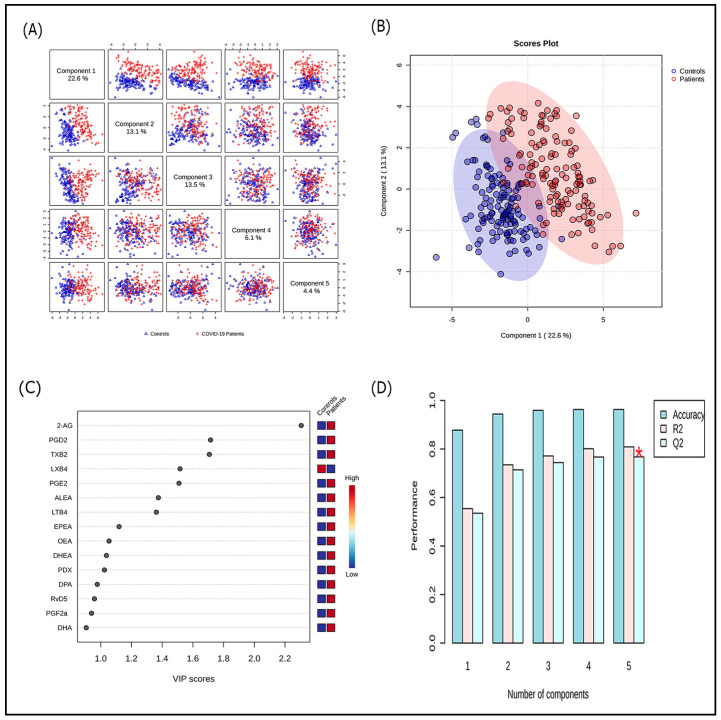
Partial least squares discriminant analysis (PLS-DA) of plasma lipid mediators between Coronavirus disease 2019 (COVID-19) patients and controls. (**A**) Overview with five first-ranked components; (**B**) PLS-DA 2D (two-component) scores plot display clustering and class discrimination of patients from controls; (**C**) Variable importance in projection (VIP) scores ranking the first 15 metabolites based on their importance in discriminating BD patients from controls. The red and blue boxes on the right indicate whether the metabolite is increased or decreased. High VIP scores indicate the metabolites that greatly discriminate COVID-19 patients from controls. A threshold of 1.5 allowed for the selection of the five most discriminant variables (i.e., 2-AG, PGD2, TXB2, LXB4, and PGE2); (**D**) Model performances according to the number of components (*, indicates the model with the best performance). 2-AG: 2-arachidonoylglycerol; ALEA: alpha-linolenoylethanolamide; DHA: docosahexaenoic acid; DHEA: docosahexaenoylethanolamide; DPA: docosapentaenoic acid; EPEA: eicosapentaenoylethanolamide; LTB4: leukotriene B4; LXB4: lipoxin B4; OEA: oleoylethanolamide; PDX: protectin DX; PGD2: prostaglandin D2; PGE2, prostaglandin E2; PGF2a: prostaglandin F2α; RVD5: resolvin D5; TXB2: thromboxane B2.

**Figure 4:**
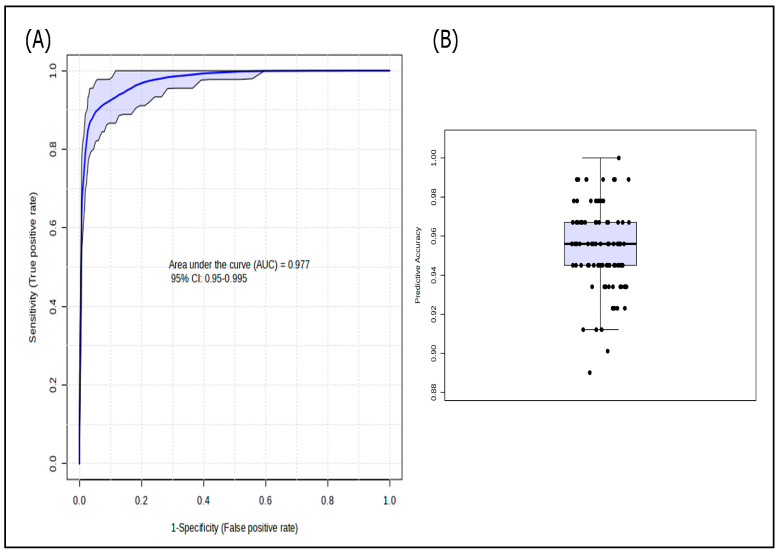
Multivariate receiver operating characteristic curve-based model evaluation of the combination of the metabolites 2-arachidonylglycerol, prostaglandin D2, prostaglandin E2, lipoxin B4, and thromboxane B2 (**A**) for the comparison between patients and controls, and (**B**) model accuracy.

PLS-DA highlighted a fair clustering between COVID-19 patients and HC. The most contributory variables in group discrimination (VIP score > 1.5) were 2-AG, PGD2, TXB2, LXB4, and PGE2. The model had a high ability to separate patients and HC with an accuracy of 95.9% (Figure 3). Multivariate ROC curve-based model evaluation, integrating the five variables with a VIP > 1.5, elected combined overexpression of 2-AG, PGD2, PGE2, and TXB2, and down-expression of LXB4 as an accurate lipidomic signature for COVID-19 [AUC (95% CI): 0.977 (0.95–0.995); median predictive accuracy of 0.95] (Figure 4).

### 3.3. Lipidomic Profile According to Disease Severity and Selected Adverse Conditions

Patients with severe COVID-19 had higher TXB2 and LTB4 levels. Moreover, AA-derived proinflammatory oxylipins (i.e., PGs, LTs, and TXs), the LTB4/LXB4 ratio, LTs/LXs ratio, and the proinflammatory/pro-resolving oxylipins ratio were noticeably higher in patients with the severe form. One hundred fifty patients had serum CRP above 50 mg/L, evocating an important inflammatory process. They showed increased levels of proinflammatory (i.e., PGD2, LTB4) and pro-resolving (i.e., 18-HEPE, 7,17-di-OH-DPA, PDX) oxylipins, as well as eCBs (i.e., OEA, ALEA, AEA, EPEA). Forty-nine patients with plasma D-dimers above 1000 µg/L, suggestive of a high prothrombotic risk, exhibited increased levels of LTB4, OEA, and ALEA (Supplementary Table S1).

## 4. Discussion

**Figure 5:**
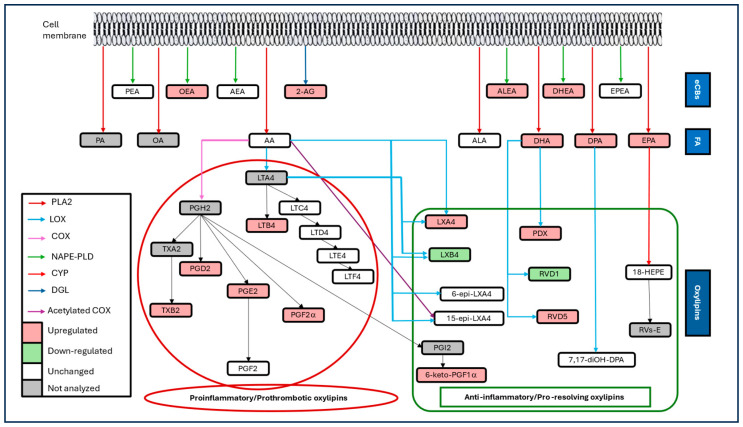
Summarized overview of metabolic pathways of the targeted lipid mediators, highlighting the pattern changes in patients with Coronavirus disease 2019 (COVID-19). 2-AG: 2-arachidonoylglycerol; 18-HEPE: 18-hydroxy eicosapentaenoic acid; AA: arachidonic acid; AEA: anandamide; ALA: α-linolenic acid; ALEA: α-linolenoylethanolamide; COX: cyclooxygenase; CYP: cytochrome P450; DGL: diglycerol lipase; DHA: docosahexaenoic acid; DHEA: docosahexaenoylethanolamide; DPA: docosapentaenoic acid; eCBs: endocannabinoids; EPA: eicosapentaenoic acid; EPEA: eicosapentaenoylethanolamide; FA: fatty acids; LOX: lipoxygenase; LT: leukotriene; LX: lipoxin; NAPE-PLD: N-acyl phosphatidylethanolamine phospholipase D; OA: oleic acid; OEA: oleoylethanolamide; PA: palmitic acid; PLA2: phospholipase A2; PDX: protectin DX; PEA: palmitoylethanolamide; PG: prostaglandin; RV: resolvin; TX: thromboxane.

This study revealed a clear shift in the lipidomic profile in COVID-19 patients, characterized by the overexpression of PUFAs, proinflammatory and pro-resolving oxylipins, and eCBs (Figure 5). Severe COVID-19 was associated with an increase in AA-derived proinflammatory oxylipins at the expense of pro-resolving oxylipins, as well as increased eCB tone. The findings corroborate the key role of these LMs in the pathophysiology of COVID-19. Therefore, targeting LM pathways could be a promising therapeutic option in this disease.

Literature has documented an alteration, generally an upregulation of LMs in COVID-19 [10,11,12,13,14,15,16,17,18,19,20,21,22,23,24]. Besides the well-known cytokine storm, the disease is accompanied by a lipid storm characterized by upregulation of PUFAs, oxylipins, and eCBs. SARS-CoV-2 causes cell death and cell debris, which provokes tissue infiltration by immune cells and activation/release of enzymes such as phospholipases, COX, LOX, CYP, hydrolases, and lipases [4].

The activated enzymes catalyse the hydrolysis of fatty acids and lysophospholipids from the cell membrane, as well as their conversion into oxylipins and eCBs, among other derivatives [32,33]. The viral infection initially induces an eicosanoid storm, characterized by the synthesis of the pro-inflammatory eicosanoids (i.e., PGs, TXs, and LTs) [34]. The mediators stimulate the production of cytokines, chemokines, and growth factors by immune cells, contributing to the cytokine storm that is responsible for COVID-19-associated inflammation, endothelial injury, and hypercoagulability [33,34]. Subsequently, high concentrations of PGD2 and PGE2 induce a lipid mediator-class switch from the 5-LOX pathway to 12-LOX/15-LOX pathways, resulting in the production of SPMs [5,35]. A default in the switch mechanism is thought to prevent the resolution of inflammation, leading to chronic inflammation and disease [5,6].

While the increase in pro-inflammatory oxylipins is plausible for an inflammatory disease like COVID-19, SPM upregulation is somewhat surprising, as inflammatory diseases are expected to show resolution defaults. However, Overexpression of SPMs is a normal response to strong inflammation. Besides producing COX and 5-LOX necessary for the synthesis of proinflammatory oxylipins, activated epithelial, endothelial, and immune cells produce 12-LOX, 15-LOX, and CYP, which are responsible for the synthesis of LXs, RVs, and PDs [5,35]. Moreover, SPM synthesis requires a prior increase in proinflammatory oxylipins. High amounts of PGD2, PGE2, and LTB4 stimulate 15-LOX, promoting the synthesis of LXs [14]. Such overexpression might be understood either as a failure or a success of the resolution. It may imply a failed attempt to counteract strong inflammation due to receptor or post-receptor defaults. Alternatively, it could signal a forthcoming resolution of inflammation and recovery. Prospective studies are needed to clarify the significance of SPM upregulation in COVID-19.

Like n3 PUFAs and oxylipins, eCBs were increased in patients, reflecting the hyperactivation of the ECS in COVID-19. Enhanced endocannabinoid tone in COVID-19 has been consistently reported in the literature [17,22,23,24]. Such an increase results in the release/activation of eCB-synthesizing phospholipases and lipases from epithelial and inflammatory cells. The synthesis of eCBs occurs on demand as an adaptive response to counteract the insult/injury and restore homeostasis [8]. Hence, ECS activation is a normal response to COVID-19-associated tissue inflammation/injury. However, whether high eCB levels are protective or maladaptive needs further elucidation. Again, prospective cohort studies are necessary to clarify the topic.

In this study, no specific LM demonstrated sufficient power to distinguish between COVID-19 patients and healthy individuals. However, the ratios PGD2:LXB4, LTB4:LXB4, 2-AG:AA, and 2-AG:AEA accurately distinguish COVID-19 patients from controls. The ratios are related to AA pathways, which involve AA itself, as well as AA-derived oxylipins and eCBs. Accordingly, most studies proved dysregulated AA pathways and altered AA-derivatives in COVID-19 [11,12,13,15,16,18,20,22,23,24]. The LTB4:LBX4 ratio reflects the efficacy of switching from active inflammation to resolution. The two oxylipins derive from the same enzymatic pathway. The enzyme 5-LOX in leukocytes forms the epoxide intermediate LTA4, which is transformed into LTB4 by 5-lipoxygenase-activating protein [FLAP] or into LXA4 and LXB4 by 12-LOX [36]. The higher ratio suggests an upregulation of 5-LOX/FLAP at the expense of 12-LOX, and a dysfunction of the switching mechanism in COVID-19. This study also defined a lipidomic signature for COVID-19, which associates increases in PGD2, PGE2, TXB2, and 2-AG and a decrease in LXB4. The ratios and signature have “very good” to “excellent” accuracy as biomarkers, but they might lack specificity and be irrelevant to everyday practice. However, they help understand the role of LMs in the pathophysiology and provide therapeutic opportunities for the disease.

Studies examining lipidomic profiles in relation to COVID-19 severity have yielded mixed results. While severe forms were consistently associated with high eCB levels [17,20,22,23], the profiles of PUFAs/oxylipins varied across studies. The latter were either increased, decreased, or unchanged in severe versus non-severe COVID-19 [10,11,12,14,15,17,19,21,37]. The discrepancy may result from differences in severity definition, sampling timing, medications, comorbidities, and ethnic-specific disparities in cellular and humoral immune responses to COVID-19 [38]. In the herein series, severe forms were associated with increased AA-derived proinflammatory oxylipins, including the abundant and potent pro-inflammatory LTB4, as well as higher LTs to LXs, and proinflammatory to pro-resolving oxylipin ratios, reflecting an upregulation of proinflammatory oxylipins at the expense of SPMs. LTB4 was also increased in patients with an important inflammatory process or high prothrombotic risk. High levels of TXB2 reflect a hyperproduction of the prothrombotic TXA2, which may contribute to hypercoagulability in severe COVID-19. A lipid storm characterized by an imbalance between proinflammatory and pro-resolving oxylipins may trigger the cytokine storm, contributing to the occurrence of a severe form. Therefore, therapeutics aiming to restore an adequate proinflammatory/pro-resolving oxylipin balance are interesting options for treating severe COVID-19.

Circulating eCBs were increased in patients with an important inflammatory process and a prothrombotic state. In agreement, the literature has provided evidence of high AEA and 2-AG levels in severe/critical COVID-19 forms [17,22]. ECS hyperactivation likely reflects a more effective response to greater tissue injury and intense inflammation. Under adverse conditions, a higher endocannabinoid tone would be necessary to restore homeostasis. However, it cannot be excluded that the activation reflects a more maladaptive response of the ECS in severe forms.

The current study has a large sample size, and potential confounders were controlled. Upon inclusion, patients were free from medication that could alter the lipidomic profile or disease course. This study is one of the few that investigated PUFAs, oxylipins, and eCBs together in the context of COVID-19, providing a better understanding of their interconnected metabolic pathways. The application of machine learning approaches allowed us to gain insight into this complex disease by developing biomarkers and a lipidomic signature. This study has limitations that should be acknowledged. The case–control design prevents ascertaining causality. This study lacks a patient control group with other infectious/inflammatory diseases, which prevents ensuring that the lipidomic changes are specific to COVID-19. It was a single-centre study involving a homogeneous population group, which prevents its generalizability. Another limitation was the reduced number of LMs and the lack of examination of synthesizing/signalling pathways. Further research addressing a large panel of LMs and their metabolic pathways while involving a control patient group with other inflammatory diseases is needed to clarify the role of these LMs and their therapeutic potential in COVID-19.

New therapeutic approaches based on modulating LM pathways could be useful in COVID-19 treatment. The effect of proinflammatory oxylipins can be reduced by inhibiting their synthetising enzymes (e.g., acetylsalicylic acid, anti-COX drugs) or signalling pathways (e.g., antileukotrienes, thromboxane receptor antagonists). Likewise, interventions that increase SPM concentrations, particularly n3 derivatives, could prove useful in resolving inflammation in COVID-19. Accordingly, a recent systematic review showed that administration of n3 fatty acids reduced mortality in hospitalised COVID-19 patients with no risk of drug-associated adverse events [39].

## 5. Conclusions

This study shed some light on an unresolved issue by demonstrating an altered lipidomic profile in patients with COVID-19. The disease is associated with a lipid storm, characterized by overexpression of PUFAs, proinflammatory and pro-resolving oxylipins, and increased endocannabinoid tone. The findings suggest that the lipidomic profile could modulate disease severity. Specifically, an imbalance of proinflammatory and pro-resolving lipid mediators could lead to severe COVID-19. The study findings further confirm involvement of LMs in the disease and provide opportunities for innovative therapeutic approaches. Targeting lipid mediator-related metabolic pathways could be an alternative/adjuvant therapeutic option in severe SARS-CoV-2 infection. Further research is needed to clarify the role of these LMs in the pathophysiology of infection and to investigate whether modulating related pathways could be a therapeutic option for COVID-19 and similar infectious/inflammatory diseases.

## Figures and Tables

**Table 1:** Main characteristics of Coronavirus disease 2019 (COVID-19) patients according to disease severity.

	COVID-19 Form		
	All Forms (n = 134)	Non-Severe (n = 49)	Severe (n = 85)
General characteristics			
Female	53 (39.6%)	16 (32.7%)	37 (43.5%)
Age, year	63.3 ± 14.1	61.5 ± 14.3	64.4 ± 13.7
Age ≥ 65 years	66 (49.3%)	22 (44.9%)	44 (51.8%)
Body mass index, kg/m^2^	28.3 ± 5.09	27.3 ± 4.37	28.9 ± 5.40
Tobacco smoking	49 (36,6%)	15 (30.6%)	34 (40.0%)
Statins use	16 (11.9%)	5 (10.2%)	11 (12.9%)
Aspirin use	12 (9.0%)	5 (10.2%)	7 (8.20%)
Anticoagulation therapy	6 (4.5%)	3 (6.10%)	3 (3.50%)
Comorbidities			
Diabetes	70 (52.2%)	22 (44.9%)	48 (56.5%)
Hypertension	80 (59.7%)	28 (57.1%)	52 (61.2%)
Obesity	39 (29.1%)	13 (26.5%)	26 (30.6%)
Cardiac disease	24 (17.9%)	8 (16.3%)	15 (17.6%)
Respiratory disease	12 (9.0%)	5 (10.20%)	7 (8.20%)
Thyroid disorder	8 (6.0%)	4 (8.20%)	4 (4.70%)
Symptoms			
Days since symptoms onset	10 ± 3.70	11.2 ± 3.54	11.1 ± 3.74
Fatigue	113 (84.3%)	36 (73.5%)	77 (90.6%) **
Cough	96 (71.6%)	34 (69.4%)	62 (72.9%)
Fever	93 (69.4%)	38 (77.6%)	55 (64.7%)
Dyspnoea	78 (58.2%)	25 (51.0%)	53 (62.4%)
Myalgia	73 (54.4%)	28 (57.1%)	45 (52.9%)
Arthralgia	53 (39.6%)	19 (38.8%)	34 (40.0%)
Chills	54 (40.3%)	20 (40.8%)	34 (40.0%)
Headache	48 (35.8%)	15 (30.6%)	33 (38.8%)
Anorexia	42 (31.3%)	15 (30.6%)	27 (31.8%)
Diarrhoea	38 (28.4%)	9 (18.4%)	29 (34.1%) *
Sweating	35 (26.1%)	14 (28.6%)	21 (24.7%)
Chest pain	24 (17.9%)	5 (10.2%)	19 (22.4%)
Nausea/vomiting	23 (17.2%)	6 (12.2%)	17 (20.0%)
Anosmia	26 (19.4%)	12 (24.5%)	14 (16.5%)
Ageusia	21 (15.7%)	7 (14.3%)	14 (16.5%)
Vertigo	16 (11.9%)	5 (10.2%)	11 (12.9%)
Initial examination			
Temperature, °C	37.2 ± 0.82	37.1 ± 0.95	37.3 ± 0.78
Fever	19 (14.2%)	6 (12.2%)	13 (15.3%)
Respiratory rate, cpm	27.1 ± 6.04	22.9 ± 4.43	28.9 ± 5.30 ***
Respiratory rate > 30 cpm	27 (20.1%)	0	27 (31.8%) ***
SpO_2_ in room air ^1^, %	88 ± 5.14	92.7 ± 2.34	85.7 ± 4.75
SpO_2_ < 88% in room air	41 (35.7%)	0	41 (58.6%) ***
PaO_2_/FiO_2_, mm Hg	230 ± 82.0	284 ± 80.2	201 ± 67.6 ***
PaO_2_/FiO_2_ < 300 mm Hg	106 (80.3%)	26 (55.3%)	80 (94.1%) ***
C-reactive protein, mg/L	170 (66–164)	93 (43–126)	126 (79–201) ***
D-dimers, ng/mL	662 (475–1283)	577 (360–909)	742 (530–1493) **

Variables are expressed in numbers (percentages), mean ± standard deviation, or median (25th–75th percentile); COVID-19: Coronavirus disease 2019; cpm: cycles per minute; SpO_2_: oxygen saturation; PaO_2_: partial pressure of oxygen; FiO_2_: fraction of inspired oxygen; ^1^: determined in 115 patients; *: *p* < 0.05; **: *p* < 0.01; ***: *p* < 0.001.

**Table 2:** Plasma levels of lipid mediators in controls and Coronavirus disease 2019 (COVID-19) patients.

	Controls (n = 135)	Patients (n = 134)	*p*-Value
Polyunsaturated fatty acids, ng/mL			
Alpha-linolenic acid	877 (497–1683)	973 (500–1579)	0.969
Arachidonic acid	613 (296–1450)	547 (343–898)	0.210
Eicosapentaenoic acid	74 (39–185)	147 (61–389)	<0.001
Docosapentaenoic acid	188 (101–378)	321 (203–513)	<0.001
Docosahexaenoic acid	476 (268–926)	780 (474–1130)	<0.001
Oxylipins, pg/mL			
Proinflammatory oxylipins			
6-keto-prostaglandin F1a	5.76 (3.38–9.94)	7.65 (4.58–13)	0.002
Prostaglandin F2a	18.0 (10.7–28.3)	27.9 (17.8–40.8)	<0.001
Prostaglandin E2	7.86 (4.41–13.1)	20.2 (10.3–47.2)	<0.001
Prostaglandin D2	8.21 (4.66–12.4)	35.4 (11.1–90.5)	<0.001
Thromboxane B2	49.6 (28.6–112)	264 (82.5–556)	<0.001
Leukotriene B4	838 (344–1878)	2850 (762–19,560)	<0.001
Leukotriene D4	8.48 (8.48–8.48)	7.12 (3.38–13.1)	0.328
Leukotriene E4	10.6 (5.25–10.6)	7.14 (2.03–13.1)	0.109
Leukotriene F4	2.79 (2.79–6.59)	4.05 (1.3–8.92)	0.324
∑ proinflammatory oxylipins	995 (516–2047)	3292 (1286–20,983)	<0.001
Pro-resolving oxylipins (SPMs)			
6-epi-lipoxin A4	2.76 (1.35–4.43)	3.63 (0.82–25.1)	0.348
Lipoxin A4	2.35 (1.36–4.34)	4.07 (0.99–25.8)	0.016
15-epi-lipoxin A4	0.47 (0.26–1.22)	0.43 (0.21–1.02)	0.075
Lipoxin B4	4.53 (2.4–7.66)	1.48 (0.74–3.03)	<0.001
Resolvin D1	3.72 (1.57–14.3)	2.72 (1.21–5.78)	0.008
Resolvin D5	4.33 (2.22–8.12)	8.15 (2.51–35.9)	<0.001
7,17-di-OH-DPA	9.41 (5.28–18.5)	11.1 (4.02–32.5)	0.389
18-HEPE	18.2 (12.9–33.4)	24.9 (7.55–69.9)	0.367
Protectin DX	2.72 (1.71–6.07)	8.35 (1.92–59.4)	<0.001
∑ pro-resolving oxylipins	54.4 (40.1–105)	87.2 (31.2–280)	0.033
Endocannabinoids, ng/mL			
2-arachidonoylglycerol	9.02 (5.90–16.6)	91.6 (32.5–258)	<0.001
Palmitoylethanolamide	2.47 (1.84–10.8)	2.92 (2.21–4.03)	0.484
Oleoylethanolamide	2.19 (1.57–2.88)	3.05 (2.24–3.57)	<0.001
Anandamide	0.76 (0.55–1.87)	0.85 (0.56–1.14)	0.575
Alpha-linolenoylethanolamide	0.042 (0.030–0.065)	0.076 (0.055–0.109)	<0.001
Eicosapentaenoylethanolamide	0.044 (0.027–0.067)	0.072 (0.049–0.109)	<0.001
Docosahexaenoylethanolamide	0.76 (0.60–0.98)	1.08 (0.84–1.36)	<0.001
∑ endocannabinoids	20.7 (12.2–34.4)	99.1 (41.4–262)	<0.001

Variables are expressed as median (25th–75th percentile). COVID-19: Coronavirus disease 2019; 7,17-di-OH-DPA: 1,17-dihydroxy docosapentaenoic acid; 18-HEPE: 18-hydroxy eicosapentaenoic acid.

## Data Availability

The data presented in this study are available on request from the corresponding author due to ethical reasons.

## Supplementary Materials

The following supporting information can be downloaded at: https://www.mdpi.com/article/10.3390/microorganisms13112622/s1. Supplementary Table S1: Differential plasma concentrations of selected lipid mediators according to disease severity and severity-related conditions.

## Abbreviations

The following abbreviations are used in this manuscript:

18-HEPE	18-hydroxyeicosapentaenoic acid
2-AG	2-arachidonoyl glycerol
AA	Arachidonic acid
AEA	Anandamide
ALA	Alpha-linolenic acid
ALEA	Alpha-linolenoylethanolamide
AUC	Area Under the Curve
COVID-19	Coronavirus disease 2019
COX	Cyclooxygenase
CYP	Cytochrome P450
DHA	Docosahexaenoic acid
DHEA	Docosahexaenoylethanolamide
DPA	Docosapentaenoic acid
eCBs	Endocannabinoids
ECS	Endocannabinoid system
EPA	Eicosapentaenoic acid
EPEA	Eicosapentaenoylethanolamide
LC-MS/MS	Liquid chromatography coupled to mass spectrometry in tandem
LMs	Lipid mediators
LOX	Lipoxygenase
LTs	Leukotrienes
LXs	Lipoxins
OEA	Oleoylethanolamide
PDs	Protectins
PEA	Palmitoylethanolamide
PGs	Prostaglandins
PLS-DA	Partial least squares-discriminant analysis
PUFAs	Polyunsaturated fatty acids
ROC	Receiver Operating Characteristic
RVs	Resolvins
SARS-CoV-2	Severe Acute Respiratory Syndrome Coronavirus 2
SPMs	Specialized pro-resolving mediators
TX	Thromboxane
VIP	Variable importance in the projection
