# Supplementary material for: COVID-19 is Associated with a Lipid Storm that Worsens in Cases of Severe Pneumonia
Source: Microorganisms. 2025 Nov 19;13(11):2622. doi: 10.3390/microorganisms13112622 (PMC12655192; doi:10.3390/microorganisms13112622)
Supplement: Supplementary file 1 [file microorganisms-13-02622-s001.zip › microorganisms-3956236-supplementary.pdf]

Supplementary Table S1. Differential plasma concentrations of selected lipid mediators according to disease severity and severity-related conditions.

**Severe COVID form**	**No (n=49)**	**Yes (n=85)**
Thromboxane B2, pg/mL	159 (70.8–330)	388 (105–608)**
∑ proinflammatory oxylipins, pg/mL	2177 (937–15354)	4165 (1467–28898)*
Leukotriene B4:Lipoxin B4 ratio	956 (493–8789)	2493 (1118–12569)**
Leukotrienes:Lipoxins ratio	169 (76.6–448)	270 (156–763)**
Proinflammatory oxylipins:SPMs ratio	44.8 (19.4–82.6)	59.7 (35.9–97.7)*
**Important inflammatory process**	**No (n=22)**	**Yes (n=112)**
Prostaglandin D2, pg/mL	18.1 (6.56 -58.6)	36.1 (12.2-93.1)*
Leukotriene B4, pg/mL	1102 (247-11418)	3315 (816-20429)*
Oleoylethanolamide, ng/mL	2.21 (1.62-3.55)	3.10 (2.35-3.58)*
Alpha-linolenoylethanolamide, ng/mL	0.058 (0.043-0.091)	0.080 (0.057-0.112)*
Anandamide, ng/mL	0.59 (0.39-0.87)	0.90 (0.62-1.21)**
Eicosapentaenoylethanolamide, ng/mL	0.052 (0.030-0.109)	0.074 (0.054-0.109)*
**High thrombotic risk**	**No (n=85)**	**Yes (n=49)**
Leukotriene B4, pg/mL	2389 (653-15575)	3366 (948-45693)
Oleoylethanolamide, ng/mL	2.86 (2.08-3.45)	3.28 (2.52-3.79)*
Eicosapentaenoylethanolamide, ng/mL	0.068 (0.049-0.096)	0.082 (0.049-0.114)*

Variables are expressed as median (25th- 75th percentile).

Severe COVID-19 was defined as oxygen saturation in room air <90%, signs of severe respiratory distress (accessory muscle use, inability to complete full sentences, respiratory rate > 30 breaths per minute), or PaO_2/FiO_2< 300 mm Hg; Important inflammatory process was defined as CRP > 50 mg/L; High thrombotic risk was defined as D-dimers above 1000 µg/L; SPMs: specialized pro-resolving mediators; *, p<0.05; **, p<0.01.
